# Parliament and the Professions

**Published:** 1920-02-21

**Authors:** 


### PARLIAMENT AND THE PROFESSIONS.

### Medical Referees.

Mr. Doyle asked the Minister of Pensions whether, in regard to War Pensions Committees, medical men can be paid for services rendered in connection with appeal cases. Sir L. Worthington-Evans replied that under the new procedure detailed in Circular 201, which was addressed to local War Pensions Committees on December 23, the services of Medical Referees are no longer required in connection with the forwarding of appeals to the Pensions Appeal Tribunals. The question of payment, therefore, does not now arise.

### Recognition of Army Nurses' Services.

In answer to a question by General Croft alleging some unequal treatment of Army nurses as regards recognition of war service, Mr. Churchill explained that all trained nurses who served under the War Office as nursing sisters on active service have, on being demobilised, received an official letter conveying thanks for their services. Recommendations for the award of the Royal Red Cross, whether at home or abroad, are in all cases made by the General Officer Commanding under whom the person recommended has served, and so far as he was aware there have been no cases in which nurses serving abroad, who have been so recommended, have not been given the decoration.

### Mission to Lepers.

An appeal is being made to raise half a lakh of rupees (£5,000 sterling) in Calcutta and Bengal for the Mission to Lepers. The appeal is receiving cordial support from Government House and the official and commercial world in Calcutta and the Bengal Presidency generally.

